# Supplementary material for: Evaluating the Dissemination and Implementation of a Community Health Worker-Based Community Wide Campaign to Improve Fruit and Vegetable Intake and Physical Activity among Latinos along the U.S.-Mexico Border
Source: Int J Environ Res Public Health. 2022 Apr 8;19(8):4514. doi: 10.3390/ijerph19084514 (PMC9025101; doi:10.3390/ijerph19084514)
Supplement: Supplementary file 1 [file ijerph-19-04514-s001.zip › ijerph-1599687-supplementary.pdf]

Supplementary Table S1. TSSC program effect on change in FV, PA MET-minutes and meeting FV, PA guideline by location (n=4751) with adjusted variables shown

Change in Total FV consumption from baseline to the last CHW visit a							
**Independent Variables Column:**	**City A (n=1251)**	**Town A (n=457)**	**Town B (n=747)**	**Rural A (n=702)**	**Rural B (n=540)**	**Rural C (n=467)**	**Rural D (n=587)**
	Adjusted mean difference in change and 95% confidence interval with p-value						
**Exposure** high vs. low	**0.63 (0.37, 0.88); p<0.0001**	**1.45 (0.71, 2.19); p<0.0001**	**0.62 (0.37, 0.88); p<0.0001**	**0.63 (0.44, 0.83); p<0.0001**	**0.71 (0.17, 1.24); p=0.0097**	**0.74 (0.24, 1.26); p=0.0041**	**0.44 (0.06, 0.82); p=0.0242**
Estimated Mean change (95% CI) *from baseline to the last CHW visit in each group*	***High: 1.28 (1.03, 1.53)*** ***Low: 0.66 (0.48, 0.83)***	***High: 2.13 (1.31, 2.95)*** ***Low: 0.68 (0.27, 1.09)***	***High: 1.15 (0.94, 1.36)*** ***Low: 0.53 (0.40, 0.65)***	***High: 1.37 (1.23, 1.50)*** ***Low: 0.73 (0.58, 0.89)***	***High: 1.62 (1.06, 2.18)*** ***Low: 0.91 (0.61, 1.22)***	***High: 2.20 (1.50, 2.91)*** ***Low: 1.45 (1.01, 1.90)***	***High: 0.99 (0.39, 1.59)*** ***Low: 0.55 (0.03, 1.08)***
**# program strategies received**	0.04 (-0.13, 0.21)	0.12 (-0.17, 0.40)	**-0.23 (-0.40, -0.07)**	0.07 (-0.14, 0.28)	0.36 (-0.11, 0.82)	**0.36 (0.23, 0.49)**	0.14 (-0.24, 0.52)
**Age**, year	0.01 (-0.001, 0.02)	0.01 (-0.01, 0.025)	0.01 (-0.001, 0.01)	0.002 (-0.004, 0.007)	0.006 (-0.007, 0.02)	0.007 (-0.008, 0.02)	-0.006 (-0.02, 0.004)
**Sex** female vs. male	0.07 (-0.24, 0.37)	**0.70 (0.09, 1.31)**	0.27 (-0.01, 0.55)	**-0.30 (-0.50, -0.10)**	0.24 (-0.17, 0.64)	0.06 (-0.32, 0.44)	-0.19 (-0.45, 0.07)
**Have insurance** yes vs. no	-0.06 (-0.31, 0.19)	-0.13 (-0.52, 0.27)	0.19 (-0.01, 0.39)	-0.18 (-0.37, 0.002)	-0.04 (-0.43, 0.34)	-0.14 (-0.57, 0.30)	0.07 (-0.18, 0.32)
**Poverty status** below vs. above FPL	**-0.31 (-0.57, -0.05)**	-0.31 (-0.86, 0.24)	-0.08 (-0.32, 0.16)	0.005 (-0.18, 0.19)	0.28 (-0.27, 0.83)	-0.65 (-1.56, 0.25)	0.29 (-0.76, 1.34)
**Meeting FV guideline at the last CHW visit b**							
	**City A (n=708)**	**Town A (n=304)**	**Town B (n=422)**	**Rural A (n=406)**	**Rural B (n=307)**	**Rural C (n=322)**	**Rural D (n=432)**
	Adjusted Odds Ratio and 95% confidence interval with p-value						
**Exposure** high vs. low	**2.04 (1.41, 2.96); p=0.0002**	1.97 (0.77, 5.06); p=0.1567	0.84 (0.38, 1.84); p=0.6636	**3.98 (2.19, 7.24); p<0.0001**	**2.40 (1.20, 4.82); p=0.0135**	**4.10 (1.36, 12.45); p=0.0125**	2.73 (0.94, 7.94); p=0.0652
**# program strategies received**	**1.05 (0.81, 1.36)**	0.85 (0.56, 1.30)	0.88 (0.58, 1.33)	0.87 (0.51, 1.50)	1.57 (0.86, 2.88)	**1.79 (1.29, 2.48)**	1.13 (0.28, 4.63)
**Age**, year	**1.01 (1.00, 1.03)**	0.99 (0.97, 1.02)	1.01 (0.99, 1.03)	1.01 (0.99, 1.03)	0.99 (0.98, 1.01)	0.99 (0.96, 1.03)	1.01 (0.97, 1.05)
**Sex** female vs. male	1.40 (0.85, 2.29)	1.63 (0.71, 3.73)	**2.75 (1.14, 6.63)**	0.72 (0.42, 1.23)	1.32 (0.76, 2.29)	1.12 (0.52, 2.41)	1.03 (0.40, 2.67)
**Have insurance** yes vs. no	0.87 (0.59, 1.30)	0.79 (0.47, 1.32)	1.15 (0.69, 1.90)	0.85 (0.52, 1.39)	1.22 (0.73, 2.05)	0.90 (0.39, 2.07)	1.26 (0.52, 3.02)

**Poverty status** below vs. above FPL	0.76 (0.51, 1.15)	0.65 (0.32, 1.31)	1.04 (0.56, 1.93)	1.25 (0.74, 2.11)	1.25 (0.59, 2.63)	0.17 (0.02, 1.48)	0.27 (0.02, 3.81)
**Change in Total PA MET-minutes from baseline to the last CHW visit c**							
	**City A (n=1251)**	**Town A (n=456)**	**Town B (n=747)**	**Rural A (n=702)**	**Rural B (n=540)**	**Rural C (n=467)**	**Rural D (n=587)**
	Adjusted mean difference in change and 95% confidence interval with p-value						
**Exposure** high vs. low	38.55 (-102.20, 179.30); p=0.5911	**897.62 (541.05, 1254.19); p<0.0001**	-171.11 (-345.97, 3.74); p=0.0551	**220.38 (65.05, 375.70); p=0.0055**	-147.53 (-345.83, 50.76); p=0.1445	290.94 (-55.44, 637.33); p=0.0995	189.59 (-122.59, 501.65); p=0.2333
***Estimated Mean change (95% CI)** from baseline to the last CHW visit in each group*	*High: 34.64 (-104.02, 173.31)* *Low: -3.91 (-103.26, 95.45)*	***High: 1409.83 (1031.81, 1787.85)*** ***Low: 512.21 (350.98, 673.45)***	*High: -122.60 (-308.31, 63.11)* *Low: 48.52 (-42.99, 140.02)*	***High: 798.06 (692.83, 903.28)*** ***Low: 577.68 (458.00, 697.35)***	*High: 8.28 (-199.60, 216.16)* ***Low: 155.81 (42.09, 269.54)***	***High: 586.94 (107.68, 1066.19)*** *Low: 295.99 (-5.86, 597.84)*	*High: -24.24 (-456.66, 408.15)* *Low: -213.82 (-706.87, 278.22)*
**# program strategies received**	**234.68 (133.51, 335.85)**	-58.88 (-197.38, 79.61)	-81.75 (-178.93, 15.42)	114.64 (-50.51, 279.79)	**313.89 (136.80, 490.97)**	23.44 (-67.99, 114.86)	-161.95 (-473.34, 149.45)
**Age**, year	-3.39 (-8.52, 1.74)	3.93 (-4.39, 12.25)	1.02 (-3.15, 5.19)	**-22.06 (-26.55, -17.56)**	-2.61 (-7.38, 2.15)	**-12.04 (-22.46, -1.62)**	-7.88 (-16.05, 0.29)
**Sex** female vs. male	-63.35 (-240.59, 113.90)	-0.70 (-293.28, 291.88)	99.68 (-62.76, 262.12)	152.60 (-3.32, 308.52)	**-169.08 (-321.62, -16.53)**	137.45 (-126.85, 401.75)	39.70 (-176.77, 256.17)
**Have insurance** yes vs. no	**241.08 (99.31, 382.84)**	-158.91 (-350.88, 33.07)	55.29 (-62.89, 173.47)	-83.04 (-228.11, 62.03)	**-161.77 (-307.20, -16.34)**	**323.18 (15.19, 631.17)**	74.29 (-126.52, 275.10)
**Poverty status** below vs. above FPL	**-176.52 (-326.36, -26.67)**	-128.35 (-392.83, 136.13)	-49.26 (-187.88, 89.35)	-145.98 (-292.60, 0.64)	200.74 (-6.47, 407.95)	296.38 (-929.50, 336.74)	592.28 (-272.11, 1456.60)
**Meeting PA guideline at the last CHW visit d**							
	**City A (n=1007)**	**Town A (n=361)**	**Town B (n=608)**	**Rural A (n=445)**	**Rural B (n=375)**	**Rural C (n=375)**	**Rural D (n=511)**
	Adjusted Odds Ratio and 95% confidence interval with p-value						
**Exposure** high vs. low	1.15 (0.73, 1.82); p=0.5382	**3.84 (1.05, 13.95); p=0.0414**	1.38 (0.56, 3.39); p=0.4903	**2.42 (1.35, 4.32); p=0.0029**	1.00 (0.47, 2.10); p=0.9923	**5.41 (1.62, 18.08); p=0.0061**	1.14 (0.42, 3.08); p=0.7933
**# program strategies received**	**3.62 (2.42, 5.40)**	0.76 (0.51, 1.15)	1.58 (1.05, 2.38)	**2.49 (1.37, 4.50)**	**3.87 (1.53, 9.79)**	0.98 (0.73, 1.33)	1.04 (0.35, 3.09)
**Age**, year	**0.98 (0.97, 0.99)**	1.01 (0.98, 1.03)	1.00 (0.99, 1.02)	**0.94 (0.93, 0.96)**	**0.97 (0.95, 0.99)**	0.96 (0.93, 1.00)	**0.97 (0.94, 0.99)**
**Sex** female vs. male	0.85 (0.49, 1.46)	1.05 (0.50, 2.23)	1.05 (0.58, 1.90)	**2.14 (1.20, 3.81)**	1.22 (0.67, 2.23)	2.38 (0.92, 6.18)	0.95 (0.45, 2.00)
**Have insurance** yes vs. no	1.18 (0.76, 1.83)	0.80 (0.48, 1.35)	**1.80 (1.14, 2.85)**	**0.57 (0.33, 0.97)**	0.74 (0.42, 1.29)	1.61 (0.56, 4.59)	0.93 (0.46, 1.89)
**Poverty status** below vs. above FPL	**0.59 (0.37, 0.93)**	0.68 (0.32, 1.46)	1.21 (0.69, 2.10)	**0.43 (0.24, 0.77)**	2.01 (0.82, 4.93)	0.07 (0.003, 1.66)	<0.001 (<0.001, >999.99)

Dependent variable: a, change in total FV consumption; b, meeting FV guideline (yes/no); c, change in total PA MET-minutes; d, meeting PA guideline (yes/no)

Abbreviations: CHW, community health worker; FV, fruit and vegetable; MET, metabolic equivalents; PA, physical activity; TSSC, *Tu Salud ¡Si Cuenta!* program.

High program exposure defined as participants having 4-5 CHW visits (3-4 follow-up visits). Low program exposure defined as participants having 2-3 CHW visits (1-2 follow-up visits). Linear regression models were performed for the mean difference in the change of FV and PA in each location. Logistic regression models were performed on all individuals who did not meet FV or PA guidelines at baseline in each location. The "estimated mean change" rows indicates the specific changes in FV and PA behavior within each exposure group and is *italicized* to demonstrate that these rows are detailing at the individual group level the mean change described in the "exposure" rows immediately above. **Bolded numbers** indicate statistically significant results ($p<0.05$).
